# Ferulated Pectins and Ferulated Arabinoxylans Mixed Gel for *Saccharomyces boulardii* Entrapment in Electrosprayed Microbeads

**DOI:** 10.3390/molecules26092478

**Published:** 2021-04-23

**Authors:** Federico Ohlmaier-Delgadillo, Elizabeth Carvajal-Millan, Yolanda L. López-Franco, María A. Islas-Osuna, Valérie Micard, Carole Antoine-Assor, Agustín Rascón-Chu

**Affiliations:** 1 Research Center for Food and Development, CIAD, A.C., Carretera Gustavo Enrique Astiazaran Rosas, No. 46, Col. La Victoria, Hermosillo 83304, Sonora, Mexico; federico.ohlmaierdc18@estudiantes.ciad.mx (F.O.-D.); lopezf@ciad.mx (Y.L.L.-F.); islasosu@ciad.mx (M.A.I.-O.); 2 IATE, INRAE, Institut Agro, University Montpellier, CEDEX 01, 34060 Montpellier, France; valerie.micard@supagro.fr (V.M.); carole.assor@inrae.fr (C.A.-A.)

**Keywords:** ferulated polysaccharide, oxidative cross-linking, laccase, yeast entrapment

## Abstract

Ferulated polysaccharides such as pectin and arabinoxylan form covalent gels which are attractive for drug delivery or cell immobilization. *Saccharomyces boulardii* is a probiotic yeast known for providing humans with health benefits; however, its application is limited by viability loss under environmental stress. In this study, ferulated pectin from sugar beet solid waste (SBWP) and ferulated arabinoxylan from maize bioethanol waste (AX) were used to form a covalent mixed gel, which was in turn used to entrap *S. boulardii* (2.08 × 10^8^ cells/mL) in microbeads using electrospray. SBWP presented a low degree of esterification (30%), which allowed gelation through Ca^2+^, making it possible to reduce microbead aggregation and coalescence by curing the particles in a 2% CaCl_2_ cross-linking solution. SBWP/AX and SBWP/AX+ *S. boulardii* microbeads presented a diameter of 214 and 344 µm, respectively, and a covalent cross-linking content (dimers di-FA and trimer tri-FA of ferulic acid) of 1.15 mg/g polysaccharide. The 8-5′, 8-*O*-4′and 5-5′di-FA isomers proportions were 79%, 18%, and 3%, respectively. Confocal laser scanning microscopy images of propidium iodide-stained yeasts confirmed cell viability before and after microbeads preparation by electrospray. SBWP/AX capability to entrap *S. boulardii* would represent an alternative for probiotic immobilization in tailored biomaterials and an opportunity for sustainable waste upcycling to value-added products.

## 1. Introduction

**Figure 1:**
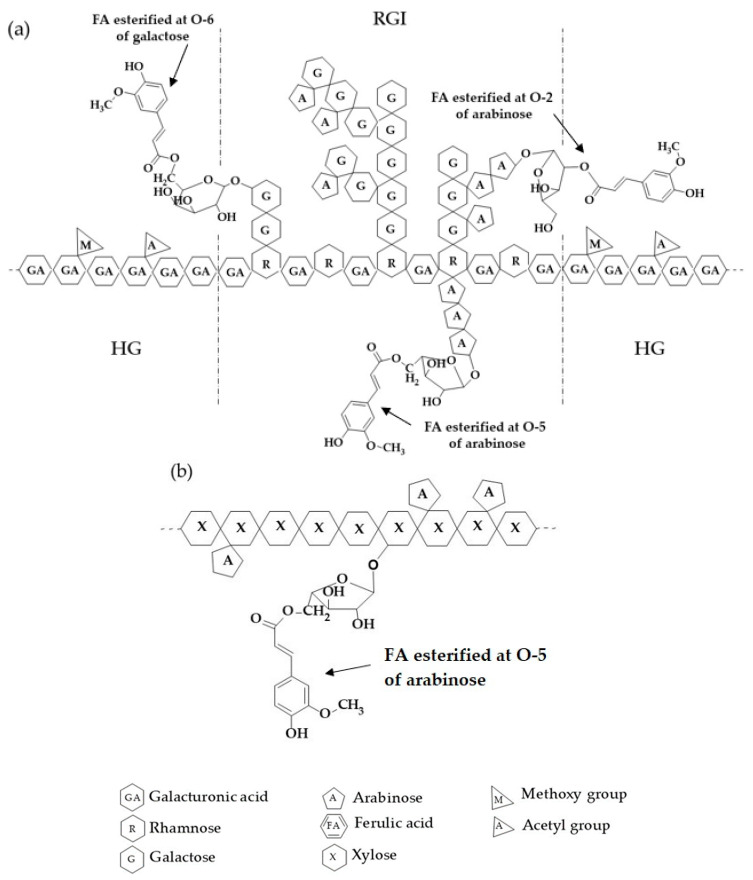
Schematic chemical structure for ferulated pectins (**a**) and ferulated arabinoxylans (**b**). HG = Homogalacturonan region, RGI = Rhamnogalacturonan I region.

Probiotic microorganisms are defined as “live microorganisms, which when consumed in adequate amounts, confer a health effect on the host” [1]. Many probiotics must reach the colon section to provide health benefits, mainly to the human gastric system. However, these microorganisms can lose viability during gastrointestinal transit due to digestive enzyme sensitiveness and low pH [2]. *Saccharomyces boulardii* (*S. boulardii*) is a probiotic yeast acknowledged and generally recognized as safe (GRAS) [3]. However, *S. boulardii* viability is damaged under stressful environmental conditions [4,5]. In this regard, technological advances such as microencapsulation have been developed to protect probiotics’ viability [6,7,8]. Microencapsulation allows bioactive materials to be coated with a single or a mixture of protective materials such as lipids, proteins, polysaccharides, sugars, and their combinations [9,10,11,12]. Electrospraying is a suitable microencapsulation method that uses an electric field to generate nano and micrometric droplets. The process involves subjecting a solution to an electric field during flow through capillary maintained at high potential; upon achieving critical value by an electric field, a jet (Taylor cone) is formed. Afterwards, the electric field causes deformation and jet distribution, resulting in fine droplets forming microparticles [13]. This technique facilitates the preparation of microparticles with tailored composition and morphology, as well as customized microstructures, for various applications [14]. Ferulated pectins and ferulated arabinoxylans are suitable for microencapsulation because they are biodegradable and biocompatible. These polysaccharides present a high potential for chemical or physical modification, which favors the achievement of suitable properties [15]. Additionally, these polysaccharides are contained in the dietary fiber of fruits and cereals already present in the human diet. They have gained great relevance due to their wide applications and health benefits. Both polysaccharides share the characteristic of having ferulic acid (FA) in their structure [16]. Traditionally, pectins are obtained from citrus peels and apple bagasse [17,18], although ferulated pectin is mainly recovered from sugar beet. Sources like sugar beet pulp, a byproduct from sugar production, have been explored. The increasing demand for sugar beet for refined sugar and bioethanol production has increased the availability of sugar beet solid waste, leading to its use as an alternative pectin extraction source [19]. Structurally, ferulated pectins consist of linear chains formed mainly by galacturonic acid units linked glycosidically by α-(1→4) bonds (homogalacturonan region, HG), with branched sections of neutral sugars (rhamnogalacturonans I and II; RGI and RGII) and other substituents. These pectins have FA bound to galactose (*O*-6) and arabinose (*O*-2 and *O*-5) residues primarily in the RG-I branching [20] (Figure 1a). Ferulated pectins may have a low or high degree of esterification, and those registering low esterification can show two gelling mechanisms, one given from galacturonic acid interactions with divalent ions (i.e., Ca^2+^) following the egg-box model [21], and the second driven by oxidative coupling of FA [22]. On the other hand, arabinoxylans are neutral nonstarch polysaccharides mainly obtained from cereals and byproducts of their industrialization, e.g., dried distillers’ grains with solubles (DDGS) from bioethanol production [23]. These polysaccharides are constituted by a linear backbone of β-(1→4)-linked xylose units which may be unsubstituted, monosubstituted with arabinose via α-(1 →3), and disubstituted with arabinose via α-(1 →3) and α-(1 → 2). FA is ester-linked to C(*O*)-5 of the arabinose residues generally attached to C(*O*)-3 of xylose units [24] (Figure 1b). Ferulated pectins’ gels induced by laccase are stable to pH changes but are not mechanically strong because RGI and RGII side chains limit the intermolecular contact [22]. Ionic pectin gels are strong but not stable to pH changes, particularly under acidic conditions [25], as their polymeric network is based on calcium bridges [26]. Arabinoxylans form stronger covalent gels resistant to changes in pH, temperature, and ionic strength [23,27,28]. However, arabinoxylans’ cross-linking can present relatively extended curing times. The formation of a mixed ferulated pectin/ferulated arabinoxylan gel has not been yet reported, despite the high application potential and attractive characteristics that could be generated in the resulting biomaterial (especially for the design of carrying matrices). Combining the two-gelling mechanism of ferulated low-esterified pectin could be a strategy to avoid the coalescence and aggregation phenomena commonly observed during the arabinoxylan microparticle preparation electrospray technique [12]. Additionally, the presence of arabinoxylan in a pectin/arabinoxylan mixed gel could bring mechanical stability to the new biomaterial. In the present work, ferulated low-esterified pectin from sugar beet solid waste (SBWP) and ferulated arabinoxylan from maize bioethanol waste (AX) were used to form a mixed gel involving two-gelling mechanisms (ionic and covalent) to entrap *S. boulardii* using electrospray, maintaining cellular viability after the microencapsulation process.

## 2. Results and Discussion

### 2.1. SBWP Characterization

The chemical composition of SBWP is presented in Table 1. The galacturonic acid content in SBWP in the present study was 52.2% (*w*/*w*), which is higher than the value (46.5% *w*/*w*) reported in a previous study for pectin from a similar industrial waste under comparable extraction conditions [29]. The galacturonic acid content in commercial pectin is approximately 60% (*w*/*w*); however, it is well documented in the literature that previous industrial processing of plant biomass might induce changes in the chemical structure of plant cell wall components, including the galacturonic acid content of pectin [25,30,31]. The neutral sugar content of SBWP is also shown in Table 1, with arabinose, mannose, and galactose being the main components. Additionally, some glucose was found in the sample. The FA content registered in SBWP (2.1 mg/g polysaccharide) was higher than some values reported in the literature for comparable pectins (0.28–1.9 mg/g) [32,33]. The FA content in SBWP was required for the oxidative coupling cross-linking mechanism in this polysaccharide.

The physicochemical characteristics of SBWP are presented in Table 2. The average molecular weight of SBWP was 468 kDa; this value is superior to some expressed in the literature (35–90 kDa), probably due to aggressive extraction conditions used in those studies [24,34]. The degree of methoxylation and acetylation of SBWP were 30% and 13%, respectively. Therefore, SBWP was classified as low-esterified and low-acetylated pectin, capable of forming calcium-induced gels. The latter is a handy feature for the fabrication of microbeads by electrospray [12]. The combination of two gelling mechanisms in this pectin allowed for particle surface stabilization by fast ionic gelation with Ca^2+^ ions and a covalent cross-linking from the inside by laccase-triggered oxidative coupling. AX used in the present study was extracted and characterized as previously described [23]. It presented a FA content of 6.46 mg/g polysaccharide, an arabinose to xylose ratio of 1:1, and a molecular weight of 200 kDa.

### 2.2. Biomass Production and Cells Viability

**Figure 2:**
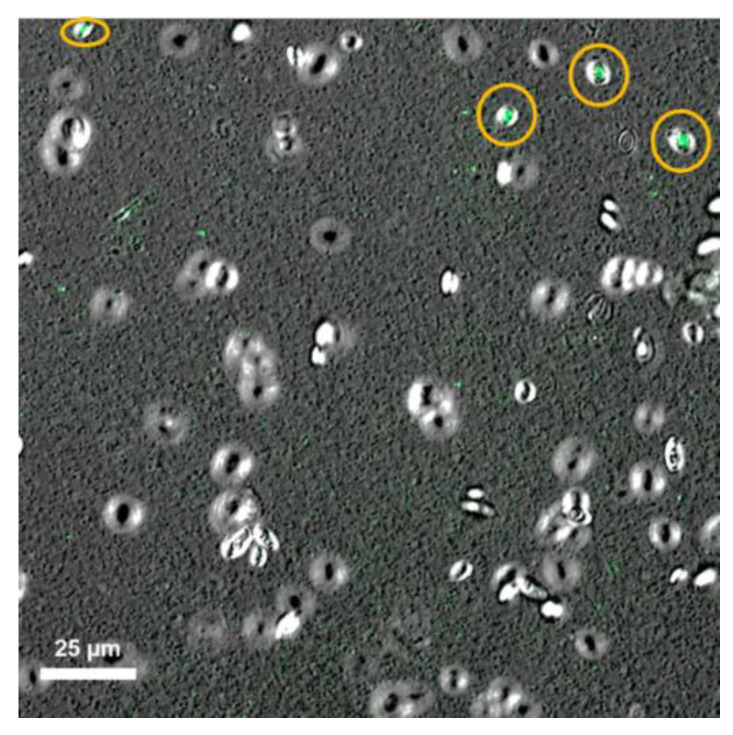
Confocal laser scanning microscopy image of *S. boulardii* fresh cells stained with propidium iodide, observed to 20× magnification applying a monoparametric detection.

After 6 h of biomass production, 2.08 × 10^8^ cells/ mL of *S. boulardii* were recovered. A fresh sample of probiotic cells was stained with propidium iodide and analyzed in a confocal laser scanning microscope, leading to viability confirmation (Figure 2). It has been reported that viable cells do not take in propidium iodide; conversely, unviable cells take this stain inside. Therefore, only unviable cells show the propidium iodide fluorescence [34].

As observed from Figure 2, cellular viability was maintained after biomass production. The color selected to note fluorescence was green. Additionally, as no viability loss was observed from several fields, an exhaustive search for some extracellular damaged cells was carried out. The only observed cells with extracellular damage are shown in Figure 2. These results confirmed that *S. boulardii* harvested after 6 h of culture under our biomass production conditions presented cellular viability.

### 2.3. Polysaccharides Gelation and Microbeads Preparation

The gelation of ferulated polysaccharides has been investigated via FA dimerisation using oxidative conditions; for instance, enzymatic free radical generating agents (i.e., laccase) [24]. Four di-FA isomers (8-5′, 8-*O*-4′, 5-5′ and 8-8′) and one tri-FA (4-*O*-8′, 5′-5″-dehydrotriferulic acid) structure have been reported in the gels formed. The 8-5′and 8-O-4′ di-FA forms are commonly preponderant in these gels [28]. In the present study, the covalent cross-linking structures (di-FA and tri-FA) were measured in SBWP/AX microbeads before and after laccase exposure (Table 3). After 2 h of polysaccharides gelation, 54% of the initial FA content in the mixture was oxidized, resulting in the formation of di-FA and tri-FA participating in the polymer´s network development. The 8-5′, 8-*O*-4′, and 5-5′ structures represented 79%, 18%, and 3% of the total di-FA in the mixed gel, respectively. It is well documented that 8-5′ and 8-*O*-4′ di-FA are the major isomers formed after oxidation reactions of FA monomers in ferulated polysaccharides [35]. Indeed, in nature, the major dehydrodimers are 8-*O*-4′ and 8-5′ for most angiosperm tissues, but abundant dimer isomers are also found in ferulated polysaccharide gels prepared under controlled conditions [36,37]. Just a small amount of tri-AF was formed in the microbeads after 120 min of laccase exposure. The di-FA + tri-FA (covalent cross-linking content) in SBWP/AX microbeads was 1.15 mg/g polysaccharide, which is high in relation to other gels based on ferulated polysaccharides recovered from agroindustrial byproducts [23,37].

**Figure 3:**
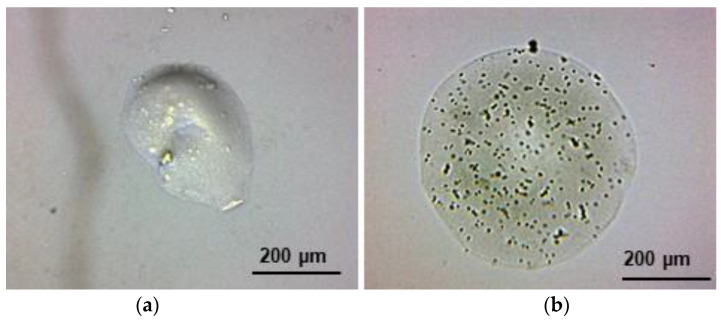
SBWP/AX (**a**) and SBWP/AX+ *S. boulardii* (**b**) microbeads images. Optical microscopy observation at 10× magnification.

SBWP/AX and SBWP/AX+ *S. boulardii* microbeads presented an average diameter of 214 ± 66 µm and 344 ± 126 µm, respectively (Figure 3). SBWP/AX+ *S. boulardii* microbeads showed a more spherical and regular shape in relation to SBWP/AX, which could be related to the yeast’s presence inside the structure. It has been reported that other microorganisms (such as *Bifidobacterium* and *Debaryomyces*) improved the AX gel morphology [37,38]. Still, this kind of behavior has not previously been reported for mixed gel or *S. boulardii*. Complementary research is needed to elucidate the mechanism by which this yeast improves the microbead structure stabilization and shape. It has been suggested that *Bifidobacterium* interacts with polysaccharides such as AX, forming biofilms via bacteria surface protein complexes [39]. An interaction *S. boulardii*–AX/pectin could be present in the present study, contributing to preserving the microbead structure, but further research is needed to test such an argument.

On the other hand, SBWP/AX and SBWP/AX+ *S. boulardii* microbeads showed no aggregation or coalescence. The fast ionic gelation of the low methoxy SBWP used in the present study stabilized the particle surface, minimizing the interlinking of polymers from one droplet to another, as previously reported for other low methoxy pectins [12]. These results indicate that combining the two gelling mechanisms present in the mixed gel system can be a strategy to avoid the coalescence and aggregation phenomena during microparticle preparation using the electrospray technique unlike previous reports where using AX with long gelation times showed high aggregation [12,37]. The microbeads surface of AX alone does not stabilize soon enough on the particle surface and may join the surrounding particles into one big bead or pearl, or into extended aggregates of particles. On the contrary, SBWP ionic interaction will stabilize the droplets’ surface. SBWP has a low degree of esterification, which allows galacturonic acid interactions with divalent ions such as Ca^2+^ to form the egg-box model [21]. SBWP and AX (in the presence of free radical-generating agents such as laccase) undergo oxidative gelation through the coupling of FA residues resulting in the formation of di-FA and tri-FA. It has been reported that the FA aromatic ring operates as a reactive site in the cross-inking mechanism of ferulated polysaccharides [24]. The FA oxidative coupling gave rise to covalent cross-linking, allowing for a stable and robust SBWP-AX covalent polymeric network inside the particle. This combination has proven adequate for electrospray fabrication of particles with neither aggregation nor coalescence, a desirable characteristic for further standardized target delivery of the immobilized cells.

**Figure 4:**
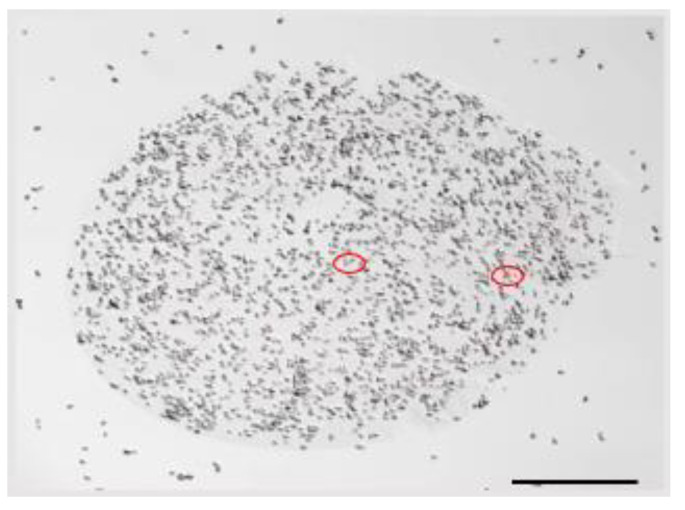
Confocal laser scanning microscopy image of SBWP/AX+ *S. boulardii* stained with propidium iodide. Circled in red two non-viable cells of *S. boulardii*. 10× magnification applying a monoparametric detection.

Figure 4 shows a confocal laser scanning microscopy image of a SBWP/AX+ *S. boulardii* microbead with yeast cells stained with propidium iodide. It can be observed that microbeads fabrication by electrospray did not considerably affect the viability of *S. boulardii* as only two nonviable cells showed the propidium iodide fluorescence (circled in red). Most particles showed fully viable cells after the electrospray conditions, exerting high voltage on the whole system. To the authors’ knowledge, this is the first report on ferulated-mixed polysaccharide gels and their application in microbeads design for immobilization of a probiotic cell.

The results presented here show this mixed gel-based material’s potential to design systems focused on carrying probiotics for food and nonfood applications. Furthermore, therapeutic compounds and functional molecules may also be trapped and carried by this system. The fine details of structural interaction between both ferulated polysaccharides is an opportunity for further research, to investigate whether their structural differences would partly restrict the oxidative coupling between pectins and arabinoxylans. Nevertheless, the microbeads fabrication method reported herein takes advantage of two gelling mechanisms, where fast ionic gelation of low methoxy SBWP with Ca^2+^ in the particle surface is combined with the strength and stability of covalent bonding inside the SBWP/AX microbead, resulting in an innovative fabrication technique. Furthermore, agrifood byproducts such as DDGS and sugar beet pulp may well constitute a further resource for gelling ferulated polysaccharides with exceptional characteristics for tailored value-added biomaterials.

## 3. Materials and Methods

### 3.1. Materials

Sugar beet solid waste (SBW) was kindly provided by a bioethanol production facility located in Sonora, Mexico. AX from maize dried distillers’ grains with solubles (DDGS) was extracted and characterized as previously described [23]. Laccase (benzenediol:oxygen oxidoreductase, E.C.1.10.3.2) from *Trametes versicolor*, and all the chemical reagents used were purchased from Sigma Aldrich (St. Louis, MO, USA). Commercial *S. boulardii* (CNCM I-745) was used as a probiotic cell.

### 3.2. SBWP Extraction and Characterization

Pectin was extracted from SBW, based on a methodology previously reported [40], with some modifications. Briefly, 150 g of dried SBW was dispersed in 1.5 L of 0.1 M HCl, and pH was adjusted to 1.5. The mixture was homogenized and heated at 85 °C for 2 h. Then the mixture was centrifuged (10,000× *g*, 15 min, 25 °C) (Thermo Scientific, Waltham, MA, USA). The supernatant was treated with ethanol in a 1:2 (*v*/*v*) ratio and the precipitate formed was dried by solvent exchange. Dried precipitate was dispersed in Milli-Q water (1:200 *w*/*w*) for 24 h with stirring, centrifuged (10,000× *g* for 15 min), filtered through 3.0, 1.2, 0.8, and 0.45 µm and precipitated by ethanol:water 1:2 (*v*/*v*) at 4 °C to obtain SBWP which was dried by solvent exchange.

The galacturonic acid content in SBWP was determined following a procedure reported [41] using high-performance liquid chromatography (HPLC) (Varian Prostar 210, Refractive Index Detector Prostar 350, Varian, Palo Alto, CA, USA), a MetaCarb H Plus column (Agilent, Santa Clara, CA, USA; 7.8 × 300 mm) and 0.001 N H_2_SO_4_ at 0.4 mL/min and 65 °C as mobile phase. Neutral sugar composition was performed by gas chromatography [42] (PerkinElmer, Clarus 580) using a high-performance capillary column (Elite 225, PerkinElmer, 30 mL × 0.32 mm ID × 0.15 µm film thickness). FA and its dimers and trimer content were analyzed by HPLC [36] using an Alltima C18 column (250 × 4.6 mm; Alltech Associates, Deerfield, IL, USA) and a photodiode array detector, Waters 996 (Millipore, Milford, MA, USA). The degree of methoxylation and acetylation was determined as previously reported [43]. Methanol and acetic acid were produced during SBWP saponification with 1 M NaOH at 4 °C for 2 h. Isopropanol was used as an internal standard. Samples were centrifuged for 10 min at 8000× *g* and 25 °C. Supernatants were neutralized before injection. Methanol, acetic acid, and isopropanol were quantified by HPLC VARIAN 500 (Varian, St. Helens, Australia) within a Refractive Index Detector on a C18 column (Superspher 100 RP-18 endcapped, Merk KGaA, 250 × 4 mm). Elution was carried out with 4 mM H_2_SO_4_ at 0.7 mL/min and 25 °C. Molecular weight (Mw) was determined using a size exclusion chromatography system coupled to a DAWN HELOS-II 8 multi-angle laser light scattering (MALLS) detector, a refractive index Optilab T-rex detector (Wyatt Technology Corp., Santa Barbara, CA, USA), and an Agilent HPLC System (G1310B Iso-Pump, G1329B autosampler, and G1314F variable wavelength detector, Agilent Technologies, Inc., Santa Clara, CA, USA). Shodex OH-pak SBH-Q-804 and 805 (Shodex Showa Denco K.K., Tokyo, Japan) columns were utilized. The software ASTRA 6.1 was used [44].

### 3.3. Biomass Production

*S. boulardii* cells were cultured in 100 mL of commercial malt extract broth (Difco, TM, pH, 5.4 at 25 °C). Cells were incubated at 31 °C and 150 rpm for 20 h (Lab-line 3540 brand). Cell counts were conducted manually every 2 h using a Neubauer Chamber in optical microscopy (Zeiss Axio Vert. A1, Carl Zeiss Microscopy, Jena, Germany) equipped with a digital camera (Axio Cam ERC 5s, Jena, Germany). Fresh cells were recuperated by centrifugation at 6000 rpm for 5 min and 4 °C [30].

### 3.4. Microbeads Preparation

**Figure 5:**
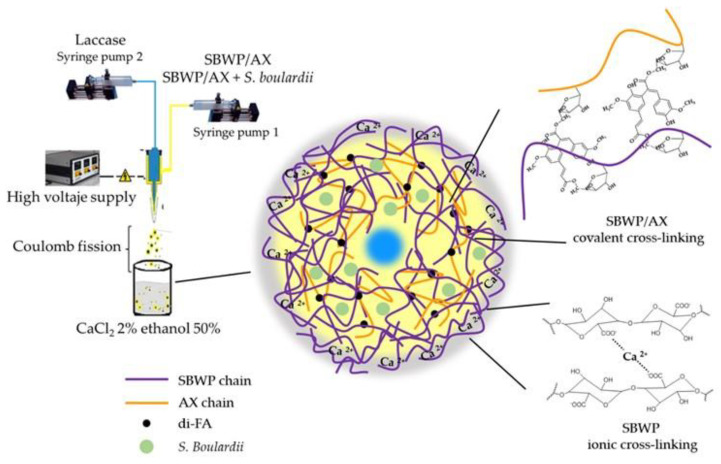
Scheme of coaxial electrospray process used to produce SBWP/AX and SBWP/AX+ *S. boulardii* microbeads.

Immobilization of *S. boulardii* inside SBWP/AX microbeads was carried out by coaxial electrospray using SpraybaseTM system (ProfectorTM, Dublin, Ireland) and two programmable syringe pumps (worldPrecision Instruments, AL-1000, Sarasota FL, USA), independently feeding a coaxial needle, as previously reported [12]. Technical electrospray conditions used were 9 kV, 0.7 mL/h for the inner needle, and 0.3 mL/h for the outer needle. SBWP/AX microspheres were prepared with and without *S. boulardii*. The outer needle conducted the mixture SBWP/AX at an overall biopolymer concentration of 53 mg/mL (40 mg/mL SBWP and 13 mg/mL AX in a 3.1 *w*/*w* biopolymer ratio) dispersed in 0.1 M sodium acetate buffer pH = 5.5. This SBWP/AX ratio ensured a 1:1 (*w*/*w*) FA contribution from each polysaccharide. SBWP/AX mixture in the outer needle was used alone or with *S. boulardii* cells (amount of cell entrapped in microspheres, 2.08 × 10^8^ cells/ mL). The inner needle conducted laccase in 0.1 M sodium acetate buffer pH = 5.5 as the cross-linking agent. Laccase dispersion contained 24 units/mg FA to ensure SBWP/AX gel-forming. The spray was received in CaCl_2_ at 2% (*w*/*v*) in ethanol:water 1:2 (*v*/*v*) fixing a distance of 7 cm from coaxial needle. It has been reported that laccase activity shows significant stability in the presence of organic solvents like ethanol, even in 50:50 *v*/*v* proportion [45]. Microbeads were stored at 4 °C. Scheme depicting coaxial electrospray process used to produce SBWP/AX and SBWP/AX+ *S. boulardii* microbeads is presented in Figure 5.

SBWP/AX and SBWP/AX+ *S. boulardii* microbeads’ morphological characteristics were investigated by optical microscopy using an inverted optical microscope (Zeiss Axio Vert. A1, Carl Zeiss Microscopy, Jena, Germany) equipped with a digital camera (Axio Cam ERC 5s, Jena, Germany). The microbeads’ average diameter was determined by using ImageJ software. FA, di-FA, and tri-FA content in microbeads were determined by high-performance liquid chromatography as described in Section 3.2 [35].

### 3.5. S. boulardii Viability

Propidium iodide was used as staining for fresh and microencapsulated cells. Cells were incubated (5 µL/mL of propidium iodide) for 10 min at room temperature. Staining was also carried out after microencapsulation to evaluate the electrospraying effect on cell viability. Monoparametric detection of propidium iodide fluorescence was performed using FL-3 (488/620 nm) confocal laser scanning microscopy (Zeiss Airyscan, Carl Zeiss Microscopy, Jena, Germany), as previously reported [34]. Briefly, viable cells do not take in the stain; conversely, unviable cells take propidium iodide inside, which reacts to DNA and stains them. Only unviable cells show the fluorescence of propidium iodide.

### 3.6. Statistical Analysis

Results are expressed as means ± standard deviation (S.D) from triplicates.

## 4. Conclusions

Microbeads based on a mixed gel of ferulated pectins and arabinoxylans were prepared by electrospray. These microbeads did not present coalescence or aggregation under the coaxial electrospray technique arrangement used. Additionally, the electrospraying conditions allowed the encapsulation of *S. boulardii* cells without losing their viability in the process. These results suggest a high potential for probiotic-loaded SBWP/AX microbeads designed for a variety of applications. Additionally, extraction of SBWP and AX from bioethanol wastes could represent a chance for sustainable byproducts’ use through upcycling to value-added products. Further studies will investigate the protection of *S. boulardii* in SBWP/AX microbeads against environmental stress such as gastric conditions.

## Figures and Tables

**Table 1:** Chemical composition of sugar beet solid waste (SBWP).

Component	Value
Galacturonic acid ^1^	52.2 ± 1.6
Rhamnose ^1^	1.50 ± 0.02
Arabinose ^1^	3.60 ± 0.04
Xylose ^1^	1.20 ± 0.02
Mannose ^1^	5.00 ± 0.04
Galactose ^1^	20.7 ± 0.4
Glucose ^1^	12.3 ± 0.2
Ferulic acid ^2^	2.1 ± 0.1

^1^ Results are expressed g/100 g SBWP dry matter. ^2^ Phenolics are expressed in mg/g SBWP dry matter. Values are presented as means ± standard deviations (*n* = 3).

**Table 2:** Physicochemical characteristics of SBWP.

Component	Value
Molecular weight (kDa)	468 ± 8
Degree of esterification (%)	30 ± 2
Degree of acetylation (%)	13 ± 2

Values are presented as means ± standard deviations (*n* = 3).

**Table 3:** Ferulic acid (FA), dimers of FA (di-FA) and trimer of FA (tri-FA) contents in SBWP/AX microbeads before (0 min) and after (120 min) laccase exposure.

Time	FA	di-FA	tri-FA
(min)	(mg/g polysaccharides)		
0	3.58 ± 0.04	0.27 ± 0.04	nd
120	1.64 ± 0.02	1.01 ± 0.20	0.14 ± 0.02

Values are presented as means ± standard deviations (*n* = 3). nd = non detected.

## Data Availability

The data presented in this study are available on request from the corresponding author. The data are not publicly available due to patent claim in process.

## Sample Availability

Samples of *Saccharomyces boulardii* and SBWP are not available from the authors.
